# A cross-sectional study of awareness of physical activity: associations with personal, behavioral and psychosocial factors

**DOI:** 10.1186/1479-5868-4-53

**Published:** 2007-11-08

**Authors:** Esther MF van Sluijs, Simon J Griffin, Mireille NM van Poppel

**Affiliations:** 1 Medical Research Council Epidemiology Unit, Cambridge, UK; 2 Department of Public and Occupational Health and EMGO-Institute, VU University Medical Center, Amsterdam, The Netherlands; 3 Body@Work, Research Center Physical Activity, Work and Health, TNO-VUmc, Hoofddorp-Amsterdam, The Netherlands

## Abstract

**Background:**

Interventions to promote physical activity frequently target hypothesized mediators of change, but these might be affected by a person's awareness of their own physical activity behavior. The paper aims to characterize a high-risk population by levels of awareness and to study associations between awareness and selected personal, behavioral and psychosocial factors.

**Methods:**

Data were collected on physical activity behavior, physical activity awareness, behavioral and psychosocial factors and anthropometry cross-sectionally at 6-month follow-up in a physical activity promotion trial. Awareness was assessed by comparing dichotomous self-rated physical activity with achieving activity levels according to international guidelines. Four groups were distinguished: 'Realistic Active', 'Realistic Inactive', 'Overestimator', and 'Underestimator'. Data were analyzed with ANCOVA, correcting for previous interventions and current physical activity level.

**Results:**

Of 632 participants (mean age: 56.3 years), 321 were inactive, 61.4% of whom rated themselves as active ('Overestimators'). Compared to 'Realistic Inactives', 'Overestimators' were older, less likely to be smokers or to intend to increase their physical activity level, and had a lower body mass index. Furthermore, 'Overestimators' had similar scores to the 'Realistic Actives' on the psychological factors, but differed significantly from the 'Realistic Inactives'.

**Conclusion:**

People who overestimate their physical activity level appear to be healthier than people who aware of their low activity level. Overestimators also scored more positively on various psychosocial factors and were also less likely to intend to change their physical activity behavior, making awareness a potential barrier in physical activity promotion. Physical activity promotion strategies might include interventions with a focus on increasing awareness in this hard to reach population.

## Introduction

Higher levels of physical activity are independently associated with various health benefits [1-4]. Yet a substantial proportion of the population in Western countries does not meet current international guidelines (i.e. engaging in moderate intensity physical activity for at least 30 minutes a day on 5 or more days of the week) [5-9]. Increasing population levels of activity therefore continues to be a major public health challenge [10]. Despite major advancements in the field, recent literature reviews on physical activity promotion suggest that there is only limited evidence for a moderately positive effect on physical activity levels and an absence of evidence for sustained changes [11-13]. There remains uncertainty about why many of these interventions are ineffective. Recent publications have started to identify groups for whom interventions might be more successful [14] and the major barriers people to behavior change. One under-explored barrier to change might be individual's lack of awareness of their own inactive lifestyle.

### Awareness of health behavior

Intention or motivation to change is one of the most important predictors of behavior change according to most of the commonly applied theoretical models [15-17]. However, whether a person intends to change his/her behavior depends A) on the belief that a change in behavior will reduce health risks and B) on the extent to which a person perceives their own behavior as 'unhealthy' [18]. For health behaviors that are clearly defined, often dichotomous behaviors such as seat belt or condom use and smoking, awareness may be a less important issue than it is for more complex health behaviors such as physical activity and diet. Studies of both fruit and vegetable intake and dietary fat intake have shown that discrepancies exist between measured and subjectively perceived consumption, especially among unhealthy individuals [19-21]. This lack of awareness also impacts on the predictive value of psychological models of behavior change, as misconception of one's own fruit and vegetable consumption has been shown to decrease the predictive value of the Theory of Planned Behavior [22].

### Awareness and physical activity behavior

Physical activity is a complex, multi-dimensional behavior and is therefore difficult to assess. It takes place in a variety of different domains; in transportation, domestic life, occupation, and recreation and is spread out over the whole day and over several days of the week. This makes evaluating the adequacy of one's own physical activity level difficult, as it requires people to reduce this complex behavior into one global index, increasing the likelihood of misclassifications. In public health research to date, awareness of physical activity levels has received little attention. Two previously published studies have reported that 57 to 67% of the general population are realistic about their physical activity level [23,24]. The studies also showed that 18 to 36% of the population overestimates their level of physical activity, which represents 48 to 61% of the currently inactive population. These unrealistic optimists (or overestimators) were on average younger and less educated than the realistic actives [25] and had a lower body mass index (BMI) than the people who were realistic about their inactive lifestyle [24]. They were also less likely to intend to increase their activity level [23,25], possibly because they saw no personal relevance in the threat of physical inactivity [18].

A lack of awareness of one's own activity level may therefore have important implications. First, it might act as a barrier to behavior change as it may result in people not seeing the need to change and hence being unaffected by public health messages concerning physical activity [18]. Second, it might also result in inactive people being overlooked or neglected in health promotion efforts as these commonly target self-rated inactive populations.

The two previous studies on this topic both included generally healthy populations. It is therefore unclear whether this lack of awareness also exists within an identified high-risk population. It might be argued that people who have been identified with a lifestyle-related disorder have different perceptions of their own behavior. It is known that a disease diagnosis makes thoughts about possible causes more salient, [26] potentially making overestimation of health behavior less likely. The data used for the analyses presented in this paper were drawn from a physical activity promotion trial including a high-risk population from general practice. Evidence from the process evaluation of this trial indicated that physical activity overestimation was one of the major barriers for general practitioners in their counseling [27]. Moreover, it was also shown that the measurements conducted as part of this trial had an impact on level of awareness of physical activity [28]. These results indicate that awareness of physical activity level is also of relevance to high-risk populations. In order to identify individuals who are unaware of their low levels of physical activity and how best to target them with health promotion interventions, there is a need to improve understanding of the personal, behavioral and psychosocial factors associated with awareness of physical activity behavior in high-risk individuals.

The aim of the current paper is therefore to study awareness of physical activity behavior in a high-risk population recruited from general practice. Individuals will be characterized according to their level of awareness and associations between awareness and personal, behavioral and psychosocial factors will be studied.

## Methods

### Study design and recruitment

This paper presents analyses of cross-sectional data collected six months after the baseline measurement of participants in a randomized controlled trial of physical activity promotion in general practice (evaluation of Physician-based Assessment and Counseling for Exercise (PACE) intervention) [29]. A Solomon four-group design using a phased double randomization was applied in the study. First, block randomization to a physical activity intervention condition or control condition was performed at general practice-level. Next, to be able to assess a possible measurement effect, individual patients were randomized within general practices [28]. Half was randomized to a group participating in the baseline and 8-week follow-up measurement, the other half was not measured at these time points. All participants took part in the assessment six months after baseline and to increase the power of the analyses, this data set was used for the current analyses. Awareness is defined as the agreement between self-rated and self-reported activity according to the current guidelines [23]. Data were collected using questionnaires and research assistants measured basic anthropometry. The Medical Ethical Committee of the VU University Medical Center in Amsterdam, The Netherlands, approved the study protocol.

Participants were recruited from twenty-nine general practices, located throughout the Netherlands. The general practitioner (GP) identified a target population meeting the following inclusion criteria: (a) being diagnosed with hypertension and/or hypercholesterolemia and/or type 2 diabetes mellitus (T2DM), (b) aged between 18 and 70 years, (c) able to be at least moderately physically active, and (d) not being in the maintenance stage of the stages of change for regular physical activity. [17] The research team subsequently randomly selected a maximum of 90 patients per practice who each received an invitation letter from their GP and a leaflet with more detailed study information. To express willingness to participate, and to check eligibility, patients were asked to return a freepost reply-card on which they indicated the average number of days on which they spent at least 30 minutes in moderate-to-vigorous physical activity in the past six months. A total of 717 participants attended a baseline visit with their general practitioner and were included in the study, all providing written informed consent.

### Measurements

All study participants were invited for the outcome assessment six months after their first visit to their general practitioner (data collected between March 2002 and January 2003). They received a postal questionnaire together with a letter inviting them to visit their general practice at a fixed period during which research assistants undertook measurements of height, weight and waist circumference using standardized methods. Participants not attending this measurement session were encouraged to return their completed questionnaire by mail and provide self-reported height, weight and waist circumference. Fifty-nine participants provided self-reported anthropometry. Analyses with independent samples t-tests did not show differences between the values for this group and the group measured by the research team (*p*-values ranging from 0.44 to 0.99), and so all participants were included in the analyses.

### Dependent variable – Awareness of physical activity level

**Figure 1:**
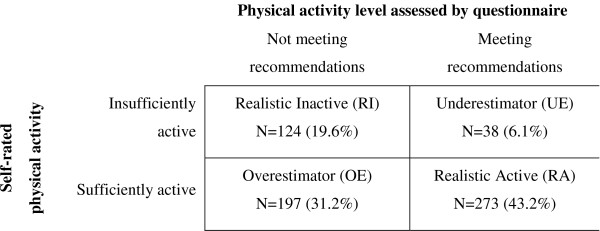
Classification of participants into categories of awareness (N = 632).

Two measures were used to assess participants' awareness of physical activity. First, to assess self-rated physical activity, participants were asked to agree or disagree (dichotomous) with the statement 'I am currently regularly physically active'. Regular physical activity was defined as performing physical activity that makes you sweat and breathe harder for at least 30 minutes a day on five or more days of the week (i.e. ACSM/CDC guideline for regular physical activity) [5]. Second, the previously validated Short QUestionnaire to ASsess Health-enhancing physical activity (SQUASH-questionnaire) [30] was used to determine whether the participant was meeting the ACSM/CDC-guideline for regular physical activity. Subsequently, self-rated and self-reported physical activity level were grouped in a 2 × 2-table, creating four groups for level of awareness: Realistic Inactives, Realistic Actives, Overestimators, and Underestimators (see Figure 1).

### Independent variables

Questionnaires were used to assess age, highest level of education, current work or educational status, smoking status and level of physical activity, using number of minutes spent on at least moderate intensity physical activity (≥ 4 MET) as assessed with the SQUASH-questionnaire. Previously validated and extensively used 5-point Likert scale questionnaires were used to assess summary scores for the following psychosocial factors hypothesized to mediate the effect of physical activity interventions [31]: self-efficacy in two domains: making time for exercise and resisting relapse [32], perceived barriers to physical activity [33], perceived benefits of physical activity [33] and cognitive and behavioral processes of change [34]. Intention to change physical activity behavior in the short (i.e. one month) or long (i.e. six months) term, was measured using the following statement with a dichotomous outcome (yes/no): 'I intend to increase my level of physical activity in the next month/6 months' [34].

### Analyses

To test for overall differences between the four groups defined by awareness, Chi Square tests were applied for dichotomous and categorical variables (intention, smoking status, gender, level of education and work status) and Analyses of CoVariance (ANCOVA) was used for continuous data. Randomization to the two intervention conditions were included as covariates in the ANCOVAs, as previous analyses had shown that the physical activity intervention had resulted in positive changes in most of the psychosocial factors [35] and that the measurement intervention (i.e. participation in the measurements) had resulted in positive changes in self efficacy for making time for exercise [28]. Furthermore, minutes of at least moderate intensity physical activity per week was also included as a covariate based on the hypothesis that it is associated with the outcome (level of awareness) and with several of the exposure measures (such as BMI and the psychosocial factors). Differences between the four groups were tested with Bonferoni post-hoc tests when applying the ANCOVA, manual comparisons using multiple Chi Square tests were used for other outcome measures. Data were analyzed in 2006 in SPSS version 12.0 for Windows, and significance level was set at a p-value less than 0.05.

## Results

### Sample characteristics

Six-month questionnaire data were available for 635 of the original 717 participants (88.6%), anthropometry data was collected on 562 (88.5% of 635) (more details on participant flow through recruitment and the study have been provided elsewhere) [28]. Three of the 635 participants were excluded because of incomplete physical activity data. No differences for age and gender were observed between those included in the analyses and those excluded. The average age of participants in the final sample was 56.3 years (SD: 9.1), 55.4% were male. Of the 632 participants, 43.2% correctly described themselves as active (RA), 31.2% overestimated their level of physical activity (OE), 19.6% correctly described themselves as inactive (RI) and only 6.1% underestimated their level of physical activity (UE) (see Table 1). Consequently, of the 321 people who were classified as inactive according to the self-reported data, 61.4% rated themselves as sufficiently active.

**Table 1:** Characteristics of participants categorized by their level of awareness (N = 632)

	**Realistic active (RA)**	**Realistic inactive (RI)**	**Overestimators (OE)**	**Underestimators (UE)**	**Differences**^a^
N	273 (43.2%)	124 (19.6%)	197 (31.2%)	38 (6.1%)	
Gender (%male)^b^	54.2	57.3	56.3	52.6	-
Age (mean, SD)^c^	56.3 (9.7)	55.1 (9.0)	57.9 (8.1)	51.8 (8.0)	RA>UE (p < 0.05) OE>RI, UE (all: p < 0.05)
Level of education^b^					-
Lower	38.8	35.2	29.1	44.1	
Middle	44.6	39.3	43.9	38.2	
High	16.5	25.4	27.0	17.6	
Work/education status^b^					-
No work	50.8	55.4	52.4	35.1	
Part time	22.7	17.4	18.3	29.7	
Full time	26.5	27.3	29.3	35.1	
Smokers (% current)^b^	17.0	31.1	20.9	21.6	RI>RA, OE (all: p < 0.05)
MVPA (min/wk)^c^					
mean (SD)	836 (639)	127 (179)	228 (230)	720 (499)	RI<RA, UE (all: p < 0.001)
Median	630	60	180	645	OE<RA, UE (all: p < 0.001)
*Anthropometry measures*					
Waist (cm)^c^	97.4 (11.9)	102.0 (13.0)	95.9 (13.2)	99.1 (12.2)	RI>OE, RA (all: p < 0.01)
BMI (kg/m2)^c^	29.0 (4.6)	30.0 (5.1)	27.9 (4.4)	28.9 (3.9)	RI>OE (p < 0.01)
Weight (kg)^c^	85.1 (15.3)	88.2 (16.5)	82.5 (16.5)	86.6 (17.4)	RI>OE (p = 0.02)

Values are means and standard deviations (SD) for continuous variables and percentages within awareness groups for dichotomous and categorical variables. SD: standard deviation; MVPA (min/wk): minutes per week of moderate to vigorous physical activity; BMI: body mass index

^a^: all statistically significant differences between groups are reported; -: no differences observed.;

^b^: tested with Chi-square;

^c^: tested with Analyses of CoVariance (ANCOVA) using Bonforoni posthoc analyses, adjusted for physical activity intervention, measurement intervention and total minutes of at least moderate intensity physical activity per week (except for outcome measure minutes of physical activity).

### Characteristics of awareness groups

There were no overall differences between the four awareness groups for gender, highest level of education and work status. The people in the Realistic Inactive group were more likely to be smokers than the Overestimators or the people in the Realistic Active group (all p < 0.05) (see Table 1). The adjusted ANCOVA analyses showed an overall association between age and awareness (F-value = 5.806, df = 3, p = 0.001). Subsequent Bonferoni posthoc analyses showed that the Overestimators and Realistic Actives were older than the Underestimators, and the Overestimators were also older than the people in the Realistic Inactive group (all p < 0.05). Levels of physical activity also differed between the groups (F-value = 99.772, df = 3, p < 0.001), showing that people in both the Realistic Inactive and the Overestimator group were less active than the other two groups (both p < 0.001), although they were not significantly different from each other. Overestimators had a lower weight, BMI and smaller waist circumference than the Realistic Inactives (all: p < 0.05). The Realistic Actives also had a smaller waist circumference than Realistic Inactives (p < 0.01). Adjustment for interventions and physical activity levels did not affect the observed associations.

### Awareness and psychosocial factors

Table 2 shows the adjusted means for the psychosocial factors by awareness groups. No overall differences between awareness groups were observed for scores on perceived benefits for physical activity, whereas statistically significant overall differences were observed for the other mediators of change (F-values ranging from 3.292 to 16.920, df = 3, p-values from 0.02 to <0.001). Post-hoc Bonferoni analyses showed that both the Realistic Inactive group and the Underestimators had higher scores for perceived barriers than the Realistic Active group and the Overestimators. Moreover, the people in the Realistic Inactive group had lower scores on both domains of self efficacy (*making time for physical activity *and *resisting relapse*) than the other awareness groups (all p-values <0.02). The Underestimators reported a higher use of the cognitive processes of change than the other groups, whereas the Realistic Inactives reported a higher use of the behavioral processes of change than the Overestimators. None of the associations were affected by adjustment for interventions or physical activity level.

**Table 2:** Adjusted means (standard error, SE), proportions and observed associations between awareness of physical activity (PA) level and physical activity-related psychosocial factors (N = 632)

	**Realistic active (RA)**	**Realistic inactive (RI)**	**Overestimators (OE)**	**Underestimators (UE)**	**Differences**^a^
N	273 (43.2%)	124 (19.6%)	197 (31.2%)	38 (6.1%)	
Benefits^b^	2.56 (0.60)	2.49 (0.62)	2.56 (0.62)	2.66 (0.55)	-
Barriers^b^	0.99 (0.56)	1.38 (0.53)	0.98 (0.53)	1.36 (0.49)	RI>RA, OE (all: p < 0.001) UE>RA, OE (all: p < 0.01)
*Self efficacy*^b^					
making time for PA	2.31 (0.97)	1.43 (0.95)	2.14 (1.02)	2.08 (1.03)	RI<RA, OE, UE (all: p < 0.01)
resisting relapse	2.35 (1.00)	1.53 (1.03)	2.27 (1.05)	2.11 (1.05)	RI<RA, OE, UE (all: p < 0.02)
*Processes of change*^b^					
cognitive	1.28 (0.68)	1.22 (0.61)	1.29 (0.61)	1.61 (0.67)	UE>RA, RI, OE (all: p < 0.05)
behavioral	1.31 (0.65)	1.13 (0.57)	1.26 (0.61)	1.47 (0.64)	RI<OE (p < 0.05)
*Intention to increase PA*^c^*(%yes)*					
in next month	17.2	23.4	15.7	34.2	UE>OE, RA (all: p < 0.05)
in next 6 months	24.2	42.7	27.4	47.4	RA<RI, UE (all: p < 0.005) OE<RI, UE (all: p < 0.05)

^a^: all statistically significant differences between groups are reported; -: no differences observed.;

^b^: tested with Analyses of CoVariance (ANCOVA) using Bonforoni posthoc analyses, adjusted for PA intervention, measurement intervention and total minutes of at least moderate intensity PA per week;

^c^: tested with Chi-square

A higher percentage of the Underestimators than the Overestimators and those in the Realistic Active group indicated that they intended to increase their physical activity within the next month and within the next 6 months. Long-term intention was also more positive for the people in the Realistic Inactive group.

## Discussion

### Characteristics of awareness groups

61.4% of the inactive population included in this study rated themselves as sufficiently active and hence overestimated their level of physical activity. A small proportion of the participants (6.1%) underestimated their level of physical activity and those were younger than the people accurately rating themselves as active and the overestimators. In contrast to previous research in the general population [25,24], in this high-risk group the overestimators were on average 1.8 years older and less likely to be current smokers than people who realistically rated themselves as inactive. In addition, they had a more favorable anthropometry profile as they had a lower weight, lower BMI and smaller waist circumference. These results suggest that in this high-risk population, overestimators were an older and apparently healthier population than the people who were aware of their inactive lifestyle. This group may have assumed that they were sufficiently active because of their more favorable health characteristics. It is common knowledge that physical inactivity is linked with weight gain and obesity, possibly leading people to conclude that not being overweight means that one is sufficiently active and that there is no need to increase activity [24]. It is interesting to see that this phenomenon is not only observed in a healthy population [23], but also in this population who are at high risk of developing cardiovascular disease due to conditions commonly associated with an unfavorable lifestyle.

Two previously published papers on awareness of physical activity reported similar proportions of overestimators within the inactive group, with figures ranging from 48% [24] to 61% [23], although these were assessed in the general population. Physical activity overestimation is of relevance to the current focus on population-level physical activity promotion, as overestimators may form a target population that is either difficult to reach or neglected in physical activity promotion efforts. One might argue that because the overestimators appear to be more healthy than the accurately inactives, they would be a less appropriate target for interventions. However, the population included in this study was already at high risk and any increases in physical activity could improve their current and future health and wellbeing [36]. Furthermore, higher levels of physical activity have been shown to be independently associated with several health benefits [2], indicating that increases in physical activity independent of weight changes are beneficial for health. Moreover, comparisons with national level data showed that the study population was on average less active and had a higher weight than the general Dutch population [37].

### Awareness and psychosocial factors

Analyses of the scores on the mediators showed that the realistic inactive group and the underestimators differed in their level of self-efficacy and use of the cognitive processes of change. However, no differences were observed between the overestimators and the realistic actives, both scoring in the direction usually associated with higher levels of physical activity [38]. Scores for both groups were different from the realistic inactives. These results suggest that perception of one's activity level might be more strongly related to these psychosocial factors than one's actual activity level. This is in line with a previous study showing that the predictive value of the TPB model for fruit and vegetable intake was greater in the realistic population than in the unrealistic estimators [22]. Importantly, the results also showed that people who considered themselves inactive (the underestimators and the realistic inactives) were more likely to intend to increase their physical activity level than the ones who considered themselves sufficiently active (overestimators and realistic actives).

Theory-based intervention strategies commonly aim to achieve behavior change by facilitating change in various psychosocial factors, i.e. mediators. Moreover, minimal intervention strategies or web-based programs usually do this by relying on self-rated behavior and reported levels of the mediators [11,39]. The results of this study indicate that targeting change in these hypothesized mediators of change in order to change physical activity levels might be ineffective without first facilitating realistic perceptions of activity levels. Providing feedback on behavior might be one way of raising awareness. Previous studies including feedback have shown that it increased intention to reduce fat intake and awareness of and intention to change vegetable intake [40]. Although in a separate study no differences in intention to change physical activity were detected after provision of immediate feedback on health indicators and fitness [41], which can be considered proxy-indicators of physical activity. The effect of providing feedback on awareness of and actual physical activity has not been studied to date, but may be an important aspect of health promotion.

### Comparing our behavior

Overestimators appear to differ from the people who accurately estimate their own level of physical inactivity. The source of this optimistic bias is unknown, although a number of possibilities have been suggested: it can result from incorrect information or other cognitive errors, but it may also have a motivation origin as it serves the need to protect self-esteem or the desire to avoid feeling afraid [18]. Self-regulation theory suggests that we are continuously evaluating our own behavior against a standard and when a discrepancy is detected this would lead to an attempt to change the behavior so that it conforms more closely to the standard [42]. However, the chosen standard for comparison might not be the correct one. As the average population levels of activity are currently fairly low [6-8], people might not perceive themselves as insufficiently active because they see themselves as more active than average, creating positive optimism in their own perceptions. This is supported by a recent study showing that overestimators tend to use downward comparison (i.e. comparing to people who are doing worse then they are) whereas realistic inactives use upward comparison [24]. Furthermore, people base the decision to change their behavior on an evaluation of the time, effort and ability required to change (e.g. 'cost' or difficulty) [18]. Together with the difficulty of evaluating the adequacy of a complex behavior such as physical activity, this might result in people opting for the 'easy' option (positive optimism) as the other option (increasing physical activity) might be evaluated as too costly. This would imply that people may try to reduce the observed discrepancy by lowering the standard for comparison instead of changing their actual behavior [42]. That is to say intentionally comparing themselves with people who are less active, potentially for reasons of self-enhancement [24].

### Study limitations

Previous research has shown that completing a detailed questionnaire on physical activity may help participants to create an overview of their physical activities throughout the week and consequently raise physical activity awareness [28]. This potential bias was controlled for as self-rated physical activity data were collected before completion of the physical activity questionnaire. However, as the data used for these analyses were collected six months into a physical activity promotion study, this measurement effect might have affected the observed prevalence. In addition, participation in the physical activity trail might have influenced the observed prevalence as well, limiting generalizibility, although the direction of this bias is difficult to assess. Despite the interventions' potential effect on the prevalence estimates, it is unlikely that these have affected the associations observed in the current analyses, which are largely in accordance with previous observations in the general population [22,24,25].

In contrast to previous studies, inclusion in the present study was based on self-reported number of days spent in at least moderate intensity physical activity in the previous 6 months. This might have led to the exclusion of both overestimators and accurately actives. The size of this potential bias is difficult to assess, but is likely to have resulted in an underestimation of the prevalence of these groups compared with an unselected group. Although a validated physical activity questionnaire was used in all studies, self-reported physical activity is known to be weakly correlated with overall energy expenditure and prone to social desirability bias [43,44]. The use of an objective measure of physical activity might overcome this issue and will potentially provide a more accurate estimate of the prevalence of physical activity overestimation.

As expected, the realistic inactives and overestimators were on average much less active than the two other awareness groups. Although not statistically significant, overestimators reported more activity than accurate inactives with more than half of the overestimators reporting more than 150 minutes of activity per week (median: 180 mins/week). This raises the question what is considered to be sufficiently active: A) achieving a total of 150 minutes of activity per week or B) being active for at least 30 minutes per day spread out over at least 5 days of the week [5]. In this study, being sufficiently active was defined according to the second definition and this was explained to the participants before their self rating of activity. Using the alternative definition would result in a lower percentage of people being classified as inactive (27.8%). The number of overestimators within this inactive group however would only be somewhat lower than we have reported, 51%. These issues highlight that the overestimators operate in a grey area between 'very inactive' and 'very active', making it difficult for them to accurately evaluate their level of activity against an arbitrary target. The target behavior should therefore be clearly defined and explained to the population in public health messages.

### Public health and research implications

Misperceptions of one's own level of physical activity might act as a barrier to behavior change interventions as it may result in people not seeing the need to change and having a low intention to change. Moreover, individuals might be overlooked or neglected in health promotion efforts as these mainly target self-rated inactive populations. In addition, self-rated behavior is commonly used as the starting point on which the content of the subsequent intervention is based, as for example in individually tailored or stage-based interventions. Misclassification of stage of change might result in mismatched interventions which are unlikely to be effective [45,46] and this should be taken into account in developing future interventions. As research on physical activity awareness has been scarce to date, many questions remain. Future work should focus on the impact of awareness on the potential to change behavior and the effectiveness of interventions, on how to effectively influence awareness and on the effects of changing awareness on both the mediators of change and on actual behavior. One suggested way of raising awareness is to encourage people to wear an objective measure of physical activity with a feedback function, such as a pedometer, providing them with immediate feedback on their behavior. There is some evidence that pedometers alone can influence behavior [47]. However, future studies need to show whether this is an effective strategy to raise awareness, whether it can be sustained and whether it improves the effectiveness of physical activity promotion interventions.

## Competing interests

The author(s) declare that they have no competing interests.

## Authors' contributions

EvS participated in the design of the study, performed data collection and data analyses and drafted and revised the manuscript. MvP participated in the design of the study and contributed to interpretation of the data and revision of the manuscript. SG contributed to interpretation of the data and revision of the manuscript. All authors read and approved the final manuscript.
